# Repellency of Silver-Gray Plastic Film on *Megalurothrips usitatus* (Thysanoptera: Thripidae) in Cowpea (*Vigna ungiculata*)

**DOI:** 10.3390/insects16101069

**Published:** 2025-10-20

**Authors:** Yuanming Chi, Yilin Zhu, Ning Nong, Zihan Zhao, Mingyue Feng, Xueyuan Cui, Maoqing Li, Yanyu Chen, Wangpeng Shi

**Affiliations:** 1 Sanya Institute of China Agricultural University, Sanya 572025, China; b20213190952@cau.edu.cn (Y.C.); sy20233193435@cau.edu.cn (Y.Z.); sy20243193635@cau.edu.cn (N.N.); sy20243193622@cau.edu.cn (Z.Z.); chenyanyu@itbb.org.cn (Y.C.); 2 Department of Entomology and MOA Key Lab of Pest Monitoring and Green Management, College of Plant Protection, China Agricultural University, Beijing 100193, China; babfeng123@163.com (M.F.); cuixueyuan1005@163.com (X.C.); lmq19972020@163.com (M.L.); 3 Institute of Tropical Bioscience and Biotechnology, Chinese Academy of Tropical Agricultural Sciences, Haikou 571101, China

**Keywords:** physical control, integrated pest management, *megalurothrips usitatus*, silver-gray plastic film

## Abstract

*Megalurothrips usitatus* (Bagnall) (Thysanoptera: Thripidae) is a serious insect pest, causing severe damage to crops in Southeast Asia and Hainan Province, China. The misuse of chemical pesticides has led to high resistance in pests to many pesticides. Physical control is an alternative method that is environmentally friendly. We investigated the effects of silver-gray plastic film on the behavior of *M. usitatus* on cowpea (*Vigna ungiculata*). The observations of behavior found that the silver-gray plastic film has different repellent effects on female and male thrips, with a repellency rate of over 90% for male thrips, while it repels female thrips at a rate of 85% in the laboratory. Field surveys have found that the number of *M. usitatus* arrived at the first peak at 7:00–9:00 a.m., and then gradually decreased, reaching its lowest point by noon, and then gradually increased to reach the second peak at 13:00–15:00 p.m. on a sunny day. In the climbing period of yardlong beans, before flowering, despite the increasing numbers of thrips, we found that the number of thrips in the silver-gray plastic film hanging area was significantly lower than that in the control area. During the early and late stages of flowering, the number of thrips in the silver-gray plastic film hanging area was significantly lower than that in the control area, but during the peak flowering period, there was no significant difference in thrips numbers between the areas with silver-gray plastic film and the control areas. These results indicate that the silver-gray plastic film is a potential method to decrease damage from *M. usitatus*, especially in the early stages of crop growth.

## Simple Summary

*Megalurothrips usitatus* is a serious crop pest in Southeast Asia and Hainan, China, and widespread pesticide use has led to resistance; a safe, environmentally friendly control is needed. The study tested whether silver-gray plastic film can alter the pest’s behavior on cowpea and reduce damage. Key findings: In the lab, the film repelled the pest, with over 90% repellency in males and about 85% in females. Field observations showed pest activity on sunny days peaks twice (7:00–9:00 and 13:00–15:00) and is lowest around noon. In yardlong beans trials, pest numbers in film-covered areas were consistently lower than in untreated areas during the early climbing stage and before/after flowering; during peak flowering the difference was not significant. The silver-gray film appears to be a promising, environmentally friendly method to reduce damage from this pest, especially in the early growth of crops. Wider use could lessen pesticide use, protect crops, and promote more sustainable agriculture.

## 1. Introduction

Thrips are important agricultural pests that adversely affect a wide range of plant species [1,2,3].Both adult thrips and their nymphs feed on the leaves, flowers, and fruits of host plants, causing substantial economic losses [4,5]. Among these pests, *Megalurothrips usitatus* (Bagnall) (Thysanoptera: Thripidae) is a prevalent flower-dwelling species known to cause extensive damage to various leguminous crops, particularly in Southeast Asia [6]. This species completes its life cycle within cowpea flowers, leading to flower drop and considerable yield reduction [7,8].

Thrips management currently relies heavily on chemical pesticides [9,10]. However, insecticidal control is challenging because thrips hide in small plant crevices and rapidly develop resistance to these chemicals [11,12,13,14]. Consequently, there is an urgent need to investigate alternative strategies to reduce thrips-related damage.

Physical control plays a pivotal role in pest management. It relies on a range of physical factors—such as light, heat, electricity, mild radiation, and mechanical devices—to suppress pest populations. Techniques include artificial capture, light and color traps, radiation-induced sterility, physical barriers, temperature regulation, and gas control [15] (Han, 2012). In the framework of integrated pest management, physical control is a safe, environmentally friendly approach that is free of chemical residues and does not promote pest resistance. These attributes align with green pest management principles and substantially enhance food safety and public health [16].

Mulch film is a key input in modern agriculture [17]. It helps maintain soil temperature and moisture, while also preserving soil’s physical and chemical properties, thereby promoting crop growth and increasing yields [18]. Recently, colored films have become more common across crops [18]. In particular, silver-gray plastic film effectively repels certain light-averse insects. For instance, in Brassica crops, silver-gray plastic film reduces *Lipaphis erysimi pseudobrassicae* (Davis) infestations [10].

*Megalurothrips usitatus* avoids light and is highly sensitive to bright conditions. The thrips are most active during overcast days, early mornings, evenings, and nights, often taking refuge on the undersides of leaves or in soil crevices to avoid light. Therefore, the use of silver-gray plastic film may effectively deter thrips infestations.

In this study, we first explored the daily activity patterns of *M. usitatus* in the field. Subsequently, we assessed the repellency efficacy of silver-gray plastic film against *M. usitatus* in a laboratory setting. Finally, we implemented the film in field trials and conducted continuous monitoring of *M. usitatus* populations.

## 2. Materials and Methods

### 2.1. Repellency Efficiency of Silver-Gray Plastic Film on M. usitatus

**Figure 1:**
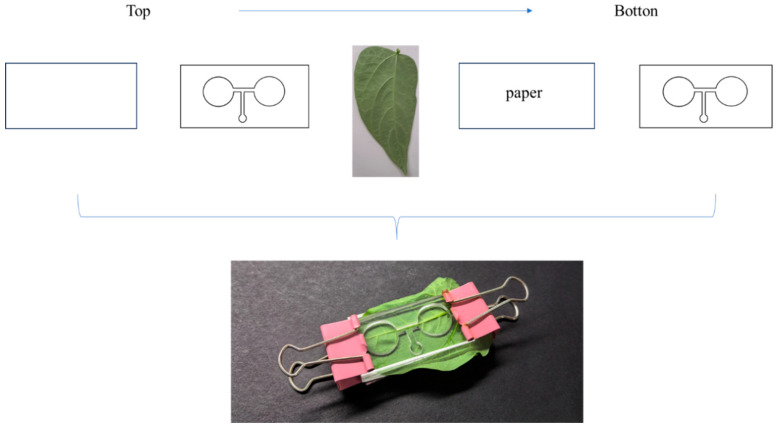
The T-shaped chamber.

The experimental device is a T-shaped chamber, adapted from the method of Quesada et al. (2012) [19]. The chamber comprises, from top to bottom, a complete acrylic plate, an acrylic plate with three circular holes, blades, filter paper, and an acrylic plate with three circular holes. The plates with circular holes measure 50 mm in length, 25 mm in width, and 2 mm in thickness. The complete acrylic plate shares these dimensions and features a 2 mm-wide channel that connects the three holes (Figure 1). Two large circular holes (15 mm in diameter) are positioned at the ends of the horizontal bar of the T. Each of these holes contains chopped cowpea fruits; however, one hole is covered by a silver-gray film. At the bottom end of the vertical stem, smaller circular holes (5 mm in diameter) serve as release points for adult thrips.

The adult thrips (4–5 d-old) were used as test insects and transferred individually into the modified Munger cells described above. Experiments on female and male thrips were carried out separately. The experiment was replicated 15 times in two blocks of three replicates. Each experiment was run for 30 min, making observations of the prey every 5 min until the prey was chosen. And each time we only used one adult thrip.

### 2.2. Insect

*Megalurothrips usitatus* used in these experiments was collected from cowpea (*Vigna ungiculata*) in Sanya, Hainan Province, China (N 18°09′34″, E 108°56′30″). The *M. usitatus* colony was maintained on Vigna unguiculata under controlled conditions in a climate chamber at (26 ± 1) °C, RH 60 ± 5%, and a 16 h light:8 h dark photoperiod, which also served as the oviposition substrate.

### 2.3. Investigation of the Daily Occurrence of Thrips

The experiment was carried out in two plots of 15 m ∗ 15 m in Hepu County, Guangxi. The experimental site was gentle slope land, latosol, 1.0%–2.5% of soil organic matter, and fertilizer and water management were consistent. The investigation was carried out during the flowering period of cowpea. Five points were selected in the southeast and northwest, and five plants were randomly selected in the field. The blue board (20 × 10 cm) was hung, and the height from the ground was 1.5 m, from 7:00 to 19:00; we counted the number of thrips on the blueboard every 1 h and then replaced the blueboard, and at each point we put 5 blue boards.

### 2.4. Field Trials

This trial was conducted by a randomized design, and it was carried out on land owned by the Sanya Institute of China Agricultural University, Sanya, Hainan, China, in 2025. This study took place from 1 January 2025, to 4 April 2025, in 4 cowpea fields of equal size (33 length × 20 width m). The temperature of the soil was a constant 25 °C ± 1 °C, the relative humidity was 60% ± 5%, and the soil pH was 6.2–7.0. The fields were surrounded by a 2.5 m high, 80 mesh net, and the soil was covered with a silver plastic film. The cowpea variety was Nan Jiang Yi Hao, which was purchased from Sanya Academy of Tropical Agricultural Sciences (Sanya, China).

The silver-gray plastic film was used in two of the fields as the treatment group, and another two were without any film as CK. The silver-gray plastic film was hung upwind in the treatment field. Each treatment group used 10 silver-gray plastic films (1.5m × 0.5m). The total number of thrips was counted (adults and nymphs).

## 3. Results

### 3.1. Repellency Efficacy of Silver-Gray Plastic Film Against Megalurothrips usitatus

**Figure 2:**
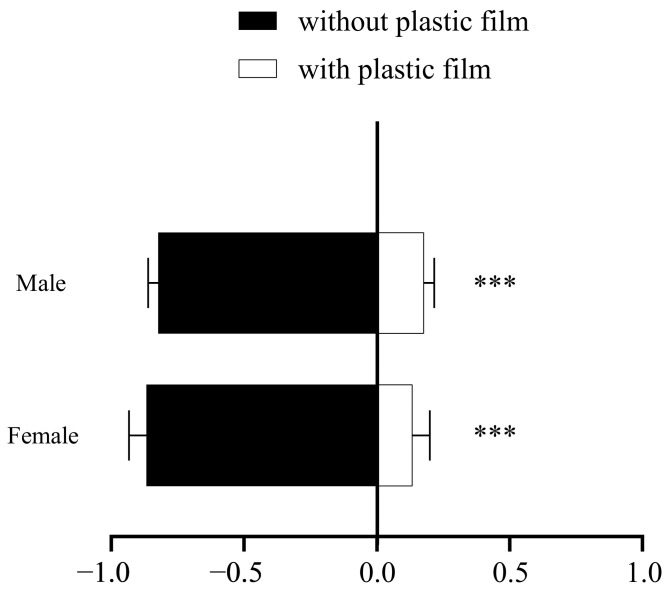
Selection probability of the bean with silver-gray plastic film and without silver-gray plastic film by *Megalurothrips usitatus* female adults and male adults (G-test) in a T-shaped chamber. Note: “***” means *p* < 0.001.

The results indicated that silver-gray plastic film has different repellent effects on female and male thrips, with a repellency rate of over 90% for male thrips, while it repels female thrips at a rate of 85%. The silver-gray plastic film significantly repels both male and female thrips (G tests. G = 9.014, *df* = 1, *p* < 0.001, and G = 6.74, *df* = 1, *p* < 0.001) (Figure 2).

### 3.2. Daily Occurrence of Thrips

**Figure 3:**
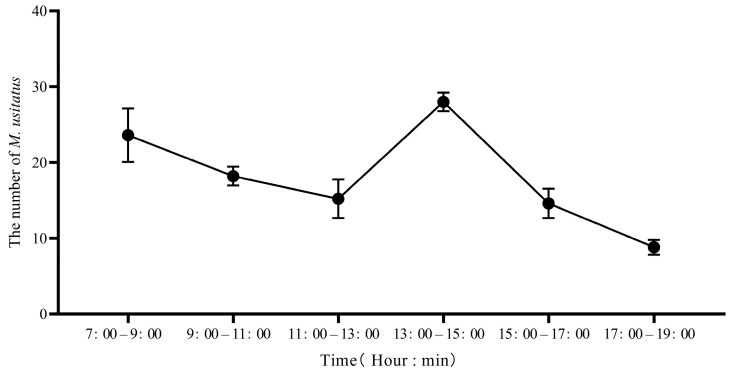
The daily occurrence of adult *Megalurothrips usitatus* at each point.

On a sunny day, *M. usitatus* activity reached the first peak between 7:00 a.m. and 9:00 a.m., followed by a gradual decline to the lowest level at noon. Activity then increased gradually, reaching a second peak between 1:00 p.m. and 3:00 p.m. (Figure 3). Consequently, these two peak periods represent the optimal times for control measures.

### 3.3. Field Trials

**Figure 4:**
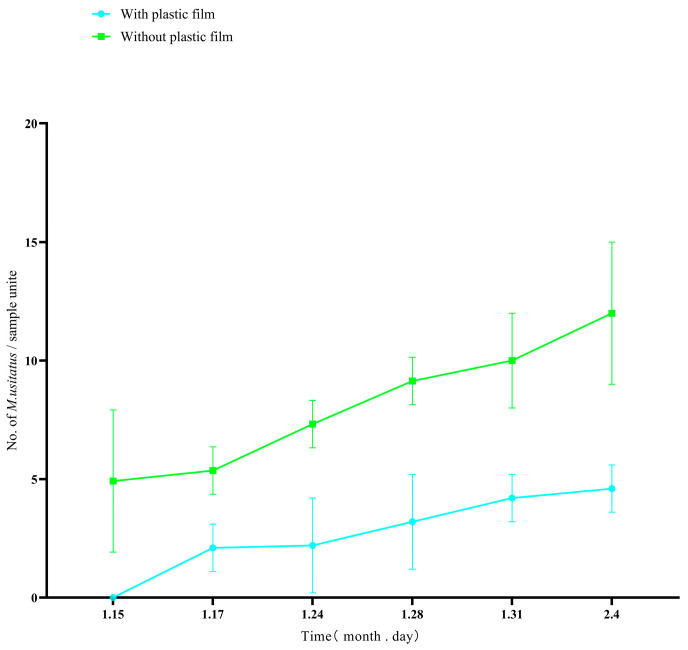
Densities of the pest thrips *Megalurothrips usitatus* in a cowpea field climbing stage treated either with silver-gray plastic film or without silver-gray plastic film.

In the climbing period of yardlong beans, before flowering, despite the increasing numbers of thrips, we found that the number of thrips in the silver-gray plastic film hanging area was significantly lower than that in the control area (Figure 4, T-test: *t* = 4.101, *df* = 10, *p* < 0.05). The results showed that before flowering, the silver-gray plastic film provides significant protection for yardlong beans.

**Figure 5:**
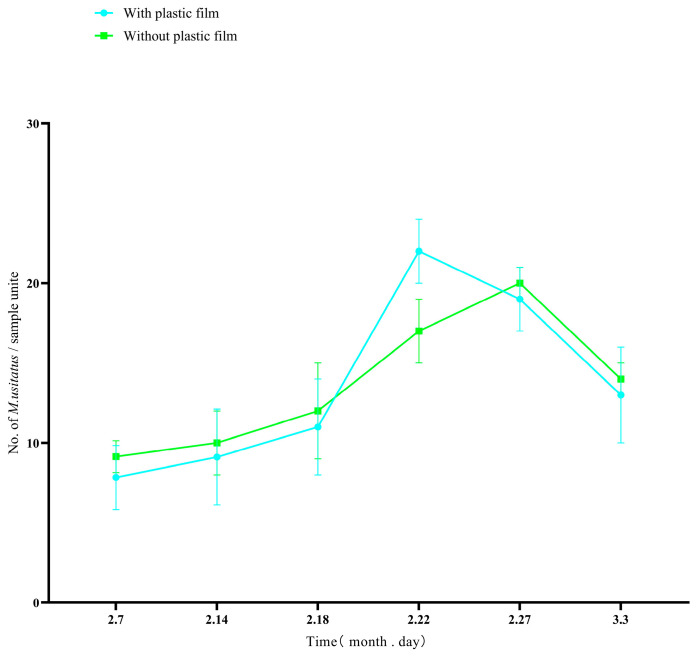
Densities of the pest thrips *Megalurothrips usitatus* in a cowpea field flowering stage treated either with silver-gray plastic film or without silver-gray plastic film.

During the early and late stages of flowering, the number of thrips in the silver-gray plastic film hanging area was significantly lower than that in the control area, but during the peak flowering period, there was no significant difference in thrips numbers between the areas with silver-gray plastic film and the control areas (T-test: *t* = 0.011, *df* = 10, *p* = 0.99) (Figure 5).

## 4. Discussion

After a long period of evolution, insects have gradually evolved complex circadian patterns in response to environmental factors such as light, temperature, humidity, food availability, competition, and predation. These adaptations enhance their survival and reproductive success. Light influences not only the expression of insect visual genes but also regulates a range of physiological processes, including biochemistry, metabolism, and secretion, which, in turn, affect growth, development, behavior, and reproduction [20,21].

*Megalurothrips usitatus*, as a negatively phototactic insect, exhibits distinct activity patterns influenced by light intensity; specifically, increased light intensity is associated with reduced activity levels. Our choice tests and field trials indicate that silver-gray plastic film effectively induces avoidance responses in *M. usitatus*. Silver-gray plastic film has been shown to deter several pest species, including *Bradysia odoriphaga* (Yang et Zhang), *Aphis gossypii* (Glover), *Tetranychus cinnabarinus* (Boisduval), and *Tetranychus urticae* (Koch) [22,23,24]. In this study, thrips density in the silver-gray plastic film area was significantly lower than in the control area during the field-climbing stage. However, during the peak flowering period, thrips densities did not differ significantly between plots with silver-gray plastic film and control plots. This pattern can be attributed to several factors. Early in plant development, cowpeas have relatively few leaves, and thrips primarily damage the growing point at the meristematic tip. The light reflected by the silver-colored film may thus deter thrips with high efficiency when the main target is the young growing point. By contrast, at flowering, thrips relocate to attack cowpea flowers, which typically appear only at full bloom. Under these conditions, the reflective light from the silver film is less effective at deterring thrips, resulting in a loss of control efficacy.

Insects frequently exhibit color preferences or aversions, with many species demonstrating a tendency to approach light [25]. *Megalurothrips usitatus* shows a strong phototactic response to blue and light-blue wavelengths [6]. In contrast to full-spectrum light, exposure to 420 nm wavelength illumination significantly prolonged the nymphal and pseudo-pupal stages, while reducing both the emergence and adult survival rates [26]. Reflectance spectroscopy reveals that silver-gray plastic film exhibits significantly higher reflectance and overall reflectivity compared to other colored mulches, particularly in the ultraviolet (380–400 nm) and yellow–green (525–607 nm) ranges [22]. This mulch film’s repellent effect on *M. usitatus* is evident from the significantly lower selection rate.

Given that thrips primarily damage the terminal growing points of stems during the climbing stage and the flowers during full bloom, the impact of silver-gray plastic film may diminish or become negligible as cowpea plants mature. Nevertheless, field conditions are complex, crops are attacked by multiple pest species, and no single control method is universally successful. Therefore, although silver-gray plastic film can effectively reduce thrips populations to some extent, its use should be integrated with other pest-management strategies to mitigate field-level damage, such as predator mites, *Beauveria bassiana* (Bals), blue boards, and aggregation pheromones [7,27,28].

## Data Availability

Datasets are available on request from the authors. The raw data supporting the conclusions of this article will be made available by the authors on request.

## References

[1] Cannon R.J.C., Matthews L., Collins D.W., Agallou E., Bartlett P.W., Walyers K.F.A., Macleod A., Slawson D.D., Gaunt A. (2007). Eradication of an invasive alien pest, Thrips palmi. Crop Prot..

[2] Diaz-Montano J., Fuchs M., Nault B.A., Fail J., Shelton A.M. (2011). Onion thrips (Thysanoptera: Thripidae): A global pest of increasing concern in onion. J. Econ. Entomol..

[3] Mouden S., Sarmiento K.F., Klinkhamer P.G., Leiss K.A. (2017). Integrated pest management in western flower thrips: Past, present and future. Pest Manag. Sci..

[4] Cloyd R.A. (2009). Western flower thrips (*Frankliniella occidentalis*) management on ornamental crops grown in greenhouses: Have we reached an impasse?. Pest Technol..

[5] Stuart R.R., Gao Y.L., Lei Z.R. (2011). Thrips: Pests of concern to China and the United States. Agric. Sci. China.

[6] Tang L.D., Han Y., Wu J.H., Li P., Fu B.L., Qiu H.Y., Liu K. (2015). Preference of *Megalurothrips usitatus* (Thysanoptera: Thripidae) to different colors and light-waves in lab. Plant Prot..

[7] Liu P.P., Qin Z.F., Feng M.Y., Zhang L., Shi W.P. (2020). The male-produced aggregation pheromone of the bean flower thrips *Megalurothrips usitatus* in China: Identification and attraction of conspecifics in the laboratory and field. Pest Manag. Sci..

[8] Tang Q.H., Li W.L., Wang J.P., Li X.J., Li D., Cao Z., Huang Q., Li J.L., Zhang J., Wang Z.W. (2022). Effects of spinetoram and glyphosate on physiological biomarkers and gut microbes in Bombus terrestris. Front. Physiol..

[9] Jensen S.E. (2000). Insecticide resistance in the western flower thrips *Frankliniella occidentalis*. Integr. Pest Manag. Rev..

[10] Reitz S.R., Gao Y., Kirk W.D.J., Hoddle M.S., Leiss K.A., Funderburk J.E. (2019). Invasion biology, ecology, and management of western flower thrips. Annu. Rev. Entomol..

[11] Akoto O., Andoh H., Darko G., Eshun K., Osei-Fosu P. (2013). Health risk assessment of pesticides residue in maize and cowpea from Ejura, *Ghana*. Chemosphere.

[12] Abtew A., Niassy S., Affognon H., Subramanian S., Kreiter S., Garzia G.T., Martin T. (2016). Farmers’ knowledge and perception of grain legume pests and their management in the eastern province of Kenya. Crop Prot..

[13] Wang Z.H., Gong Y.J., Jin G.H., Li B.Y., Chen J.C., Kang Z.J., Zhu L., Gao Y.L., Reitz S., Wei S.J. (2016). Field-evolved resistance to insecticides in the invasive western flower thrips *Frankliniella occidentalis* (Pergande) (Thysanoptera: Thripidae) in China. Pest Manag. Sci..

[14] Kirk W.D.J. (2017). The aggregation pheromones of thrips (Thysanoptera) and their potential for pest management. Int. J. Trop. Insect Sci..

[15] Han Z.J. (2012). General Theory of Plant Protection.

[16] Sang W., Gao Q., Zhang C., Huang Q.Y., Lei C.L., Wang X.P. (2022). Researches and applications of physical control of agricultural insect pests in China. J. Plant Prot..

[17] Kasirajan S., Ngouajio M. (2012). Polyethylene and biodegradable mulches for agricultural applications: A review. Agron. Sustain. Dev..

[18] Steinmetz Z., Claudia W., Miriam S., Christian B., Jan D., Josephine T., Katherine M., Oliver F., Gabriele E.S. (2016). Plastic mulching in agriculture. Trading short-term agronomic benefits for long-term soil degradation?. Sci. Total Environ..

[19] Quesada P.C.P., Schausberger P. (2012). Prenatal Chemosensory Learning by the Predatory Mite *Neoseiulus californicus*. PLoS ONE.

[20] Shibuya K., Onodera S., Hori M. (2018). Toxic wavelength of blue light changes as insects grow. PLoS ONE.

[21] Kim K.N., Huang Q.Y., Lei C.L. (2019). Advances in insect phototaxis and application to pest management: A review. Pest Manag. Sci..

[22] Wang C.Y., Qiu Y.X., Li Z.P., Zhao H.P., Wang Y., Xue M. (2021). Effect of different color plastic film on occurrence of *Bradysia Odoriphaga* in garlic field. North. Hortic..

[23] Li Z.P. (2020). Effects of Colored Film Mulching on the Occurrence to Two Species Tetranychus and Peanut Growth. Master’s Thesis.

[24] Greer L., Dole J.M. (2003). Aluminum foil, aluminium-painted, plastic, and degradable mulches increase yields and decrease insect-vectored viral diseases of vegetables. Horttechnology.

[25] Sang W., Huang Q.Y., Wang X.P., Guo S.H., Lei C.L. (2019). Progress in research on insect phototaxis and future prospects for pest light-trap technology in China. Chin. J. Appl. Entomol..

[26] Jin H.F., Lu R.C., Gong X.Y., Li F., Yang L., Wu S.Y. (2022). Effects of different wavelengths of light on growth and development of Asian bean thrips *Megalurothrips usitatus*. J. Plant Prot..

[27] Camara I., Cao K.L., Sangbaramou R., Wu P., Shi W.P., Tan S.Q. (2022). Screening of *Beauveria bassiana* (Bals.) (Hypocreales: Cordycipitaceae) strains against *Megalurothrips usitatus* (Bagnall) (Thysanoptera: Thripidae) and conditions for large-scale production. Egypt. J. Biol. Pest Control..

[28] Chi Y.M., Yu C., Feng M.Y., Shu K., Zhu Y.L., Shi W.P. (2024). Effects of field releases of *Neoseiulus barkeri* on *Megalurothrips usitatus* abundance and arthropod diversity. Sci. Rep..

